# Application of Logistic Regression and Random Forests to Assess the Relevance of Chrononutrition Information for Prediction of Overweight in Adults: Evidence from the INRAN-SCAI 2005-2006 Italian Nutrition Survey

**DOI:** 10.3390/nu18101574

**Published:** 2026-05-15

**Authors:** Karolina Bartoszek, Suzana Almoosawi, Luigi Palla

**Affiliations:** 1 Department of Public Health and Infectious Diseases, University of Rome La Sapienza, 00185 Rome, Italy; karolina.e.bartoszek@gmail.com; 2 School of Health Sciences, University of East Anglia, Norwich NR4 7TJ, UK; s.al-moosawi@uea.ac.uk

**Keywords:** chrononutrition, overweight, timing of eating, nutrition survey, prediction, logistic regression, random forests

## Abstract

**Background/Objectives**: Obesity represents a growing public health concern worldwide. Chrononutrition, a research field examining the timing and regularity of food intake, has been shown in animal models to influence body weight regulation and obesity-related outcomes. Previous research has also explored the association between chrononutrition information and BMI. Using INRAN-SCAI 2005/2006 adult nutrition data based on 3-day diet diaries (*n* = 2312), this study aims to assess whether chrononutritional information on the distribution of energy intake during the day is able to improve prediction of overweight status (BMI > 25 kg/m^2^), compared to information on energy from the whole day alone. **Methods**: This research investigates it using logistic regression and random forest models. For both types of models, three different specifications were compared: a model trained on the mean and irregularity of calorie intake over 3 days for 6 day-time intervals (MI6); a model trained on repeated measures over 3 days of calorie intake from the same 6 time intervals (RM); and a model trained on mean and irregularity of calorie intake over 3 days for the whole day (MID). The performance of the models was compared using risk prediction metrics and ROC curves. **Results:** When including additional demographic and behavioural predictors beside the energy variables, the results only showed a statistically significant difference in the performance of the logistic regression models if they were trained and tested on the same data. The models trained using chrononutrition information performed better, but the difference in diagnostic accuracy was very small (AUC = 0.7909 for MI6, *p* = 0.0086; 0.7923 for RM compared to 0.7850 for MID, *p* = 0.0072) and possibly attributable to overfitting, as it was no longer significant in the comparison within a testing set (70% training and 30% testing samples). For the random forest models, no significant difference was found. In the same models including only the energy variables, the improved performance of MI6 and RM was significantly better than for MID also in the test set (respectively, *p* = 0.0001 and *p* = 0.0002), and the gap in AUCs became substantial (AUC = 0.622 for MI6, 0.618 for RM and 0.507 for MID), indicating that socio-demographic and behavioural variables encapsulate information on energy intake by time of the day. Typical under-reporting bias present in nutritional epidemiology and the cross-sectional nature of the sample based on 3-day diaries may have affected these results, although use of diet diaries should minimize recall bias. **Conclusions:** In conclusion, the impact on health of timing and regularity of calorie intake in the day may act through other mechanisms than via overweight and may be captured by other demographic and behavioural variables; larger and prospective longitudinal studies are warranted to thoroughly investigate the added value of time-of-day information.

## 1. Introduction

Despite recent advances in the treatment and management of overweight and obesity, including the introduction of effective pharmacotherapies such as GLP-1 receptor agonists and related incretin-based agents, overweight and obesity remain among the most prevalent and persistent public health challenges worldwide [1], with substantial negative impact on health-related quality of life [2].

Against this backdrop, understanding behavioural and lifestyle determinants of weight, particularly eating behaviours, remains critical for informing effective prevention and management strategies. Beyond dietary composition alone, observational evidence indicates that the timing, distribution, and regularity of energy intake across the day relate to adiposity and weight status [3,4,5,6,7].

This concept is central to the rapidly developing field of chrononutrition, which examines the interplay between meal timing, frequency, and regularity on the one hand, and metabolic outcomes on the other hand [8,9]. Interventions such as intermittent fasting and time-restricted eating utilise temporal modulation of food intake to influence appetite regulation, glucose metabolism, and energy balance [3,10], and may have implications in the development of dietary strategy to manage weight. Chrononutrition builds on the understanding that the human circadian system, an endogenous clock averaging just over 24 h and strongly influenced by light exposure, regulates numerous physiological and metabolic processes [3,11]. Consistent with this, both observational and experimental research indicates that late eating, irregular eating patterns, and prolonged eating windows are associated with higher BMI and adverse metabolic outcomes [5,6,7,8,9,12,13]. Circadian physiology also suggests that identical meals consumed at different times of day can lead to divergent metabolic responses due to phase-dependent physiology [14]. Moreover, molecular research demonstrates that feeding rhythms interact with circadian clocks to regulate metabolic pathways, providing plausible mechanisms for these time-of-day effects [3,15].

Additional studies further show that meal timing, distribution across the day, and day-to-day regularity are associated with BMI and weight-related outcomes [5,6,12]. So far the published literature on chrononutrition has largely investigated associations that highlight potential causal relationships between eating behaviour/dietary habits and overweight/obesity underpinned by the biological mechanisms described earlier;however to our knowledge, no study to date has evaluated whether detailed chrononutrition-related information, including meal distribution and regularity across days provides added predictive value for determining an individual’s overweight or obesity status beyond total daily energy intake alone, with or without inclusion of socio-demographic or behavioural predictors.

The data used to conduct our study are provided by a national nutritional survey, namely, the INRAI-SCAI 2005-06. This cross-sectional survey was conducted through repeated food intake diaries, collected over three consecutive days on a representative sample of the Italian population [16].

The objective of this study is to assess the relevance and impact of chrononutrition information contained in diet diaries, a dietary assessment tool that records the time of intake, for predicting overweight status, using classic logistic regression models and more complex machine learning random forest models. The performance of both of them was assessed using receiver operating characteristic (ROC) curves and classification metrics, including specificity, sensitivity, positive predictive value (PPV), negative predictive value (NPV), and misclassification rate.

## 2. Materials and Methods

### 2.1. Study Design

The data used in this study were derived from the Italian National Consumption Survey INRAN-SCAI 2005-06 [16].

The survey examined 1300 randomly selected households, from geographical sections of the Italian territory. The survey had 3323 subjects in total; for the analysis in this study, only the data on adults (age 18 to 64 inclusive) were used (*n* = 2313). For the analysis carried out in this study, one unit, which had missing BMI, was removed (final *n* = 2312).

The survey consisted of self-reported (by the participants) food consumption diary for 3 consecutive days [16]. In addition, socio-demographic data of the participants were collected, such as sex, Italian area of residence, education level, profession, and employment status. The survey also contained questions about lifestyle, including alcohol intake, smoking, whether they regularly eat breakfast, means of transportation they use, hours spent on sports during the week, and number of hours during the day in which they practice some kind of physical activity. Further details on the survey can be found in the article by C. Leclercq et al. (2009) [16].

The energy intake data from the survey was divided into 6 time slots, according to the timing of the three main meals in Italy and the intervals between those meals [2]. The resulting time frames were 6 a.m.–9 a.m., 9 a.m.–12 p.m., 12 p.m.–3 p.m., 3 p.m.–7 p.m., 7 p.m.–10 p.m., and 10 p.m.–6 a.m. The irregularities of the 6 time intervals were based on the ones calculated by Palla and Lopez Sanchez (2023) [2], as well as the irregularity index for the whole day, using the following formula [17].

$$Irregularity=\frac{100\times \sum |x_{i}-\bar{x}|}{3\times \bar{x}}$$

### 2.2. Types of Models and Validation Approaches

This study made use of two predictive methods, namely, logistic regression and random forests, to predict whether a subject is overweight. The models were made to predict overweight, rather than obesity, because the number of obesity cases was relatively low—only 176 (7.6% of adults) cases observed—compared to 809 (35% of adults) cases of overweight.

For both of these methods, models for 3 different sets of energy data were generated: (1) where means and irregularity (across the 3 days of the survey) of the 6 time slots were included in the models (MI6, resulting in 12 chrononutritional predictors); (2) where repeated measures of energy intake, across the 3 days were used as predictors in the models again for all 6 time slots (RM, resulting in 18 chrononutritional predictors); (3) where a whole day mean and irregularity of energy intake across the 3 days were considered in the model as predictor, i.e., without partitioning into time slots (MID resulting in 2 nutritional predictors).

The logistic regression models tested the performance of the 3 types of model described above (a) by training the models on 100% of the data sample and testing the model on the same complete sample; (b) by training the models on 70% (chosen at random) of the data sample and testing them on the remaining 30% of the data sample; (c) by applying 10-fold cross-validation.

### 2.3. Variable Selection

The non-energy (i.e., socio-demographic and behavioural) variables for the logistic regression models were derived by stepwise forward selection, using the “step” function in R version 4.4.2 [18] by R foundation (Vienna, Austria). The optimal variables for all three conditions were chosen to minimize the AIC value of the models. The final set of variables was the union of the variables that were optimal for either of the three individual models. The variables that ended up being included in the model were hours of sport per week, age, sex, marital status, smoking status, the administrative region, use of dietary supplements, following a special diet, having been on a restrictive diet in the past year, and the level of education. Additional recorded variables, not selected through forward stepwise selection for any of the models, were area type (rural/urban), alcohol drinking, eating breakfast, the duration of following a special diet, consumption of fortified products, sedentary hours in a day, employment status, profession, hours of sport training during the week, hours of light physical activity (e.g., walking cleaning) daily, and the geographical area of Italy (North-East, North-West, Centre, and South/Islands). As stepwise forward selection may increase type 1 error/lead to overfitting, we also explored variable selection by the “lasso logit” function in Stata 18 by StataCorp (College Station, TX, USA), which led to the inclusion of all socio-demographic/lifestyle predictors in the survey, i.e., the default approach taken also for random forests models.

### 2.4. Discriminatory Accuracy Comparisons Across Models

The performance of the resulting model was checked with the “performance” library in R version 4.4.2 [19], which granted the following: posterior predictive check, collinearity check, and search for influential observations.

After the variables were chosen and the performance check did not show any major issues, the models (1–3) were fitted using all 3 variations of training and testing methods (a–c).

For each of the training and testing methods, models (1–3) were compared on their specificity, sensitivity, negative predictive value (NPV), positive predictive value (PPV)and misclassification rate. For each of these models, two probability cut-off points were chosen to classify whether the subject was overweight or not. One was simply the midpoint *p* = 0.5, and the second one was the optimal cutoff point, which jointly maximized specificity and sensitivity computed using the Youden index and implemented via the “cutpt” module in Stata [20].

For all of the models, except the cross-validated ones, ROC curves were generated and the model types compared in pairs, using the “roccomp” command in Stata [21]. The ROC curves for the cross-validated models were obtained using the “cvauroc” command [22], from which the cross-validated logistic models themselves were also generated.

### 2.5. Random Forests Model Specification and Comparisons

The random forest models were generated using the “rforest” plugin in Stata [23]. The models were built on 500 trees, and the number of variables to investigate at each split was set to the square root of the number of independent variables for each model. The depth of the forest was left unlimited, and the minimum number of observations to include at each leaf was set to 1, which allowed for overfitting when the models were tested on the same data they were trained on. Hence, they were trained on 70% (chosen at random) and tested on the remaining 30% of the data. The variables included were all those available in the survey due to the non-parametric nature of random forests, which overcomes the estimation issues linked to having a large number of predictors. The models were compared on the same metrics as the logistic regression models: specificity, sensitivity, NPV, PPV and error rate, at two cutoff points one being 0.5 and the second one an optimal cutoff point calculated using the Youden index with the “cutpt” module for Stata [20]. The ROC curves for the 3 types of model were also compared, in pairs, using the “roccomp” command from Stata [21]. Variable importance indices were also calculated.

Finally, to better understand and interpret the findings, we also repeated the comparison of the predictive models 1–3 with the same methodology, but based on energy intake predictors only, for both logistic and random forest models.

## 3. Results

### 3.1. Results from the Logistic Regression Models

#### 3.1.1. Logistic Regression Models Trained and Tested on 100% of the Data Sample

The optimal cutoff points (Table 1) for the logistic regression models trained and tested on 100% of the data sample were the following: 0.29 for MI6; 0.35 for MID; and 0.36 for RM. For the 0.5 cutoff point (Table 2), the model with repeated measures in 6 time intervals (RM) obtained the best classification metrics, the MI6 model followed, and the MID model, which did not consider chrononutrition information, performed the worst. Despite being consistent across metrics, all of the differences were very small (around 1%). At the optimal cutoff point, RM also performed the best, except for sensitivity, which was the lowest out of the three models (70.6% for RM, 80.7% for MI6, 71.1% for MID), and NPV, which was again the highest for MI6 (86.3% compared to 82.5% for both MID and RM).

**Figure 1:**
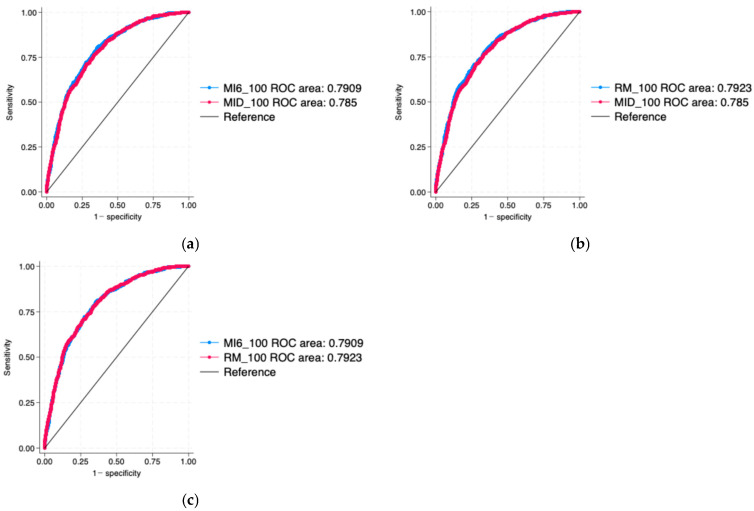
Pairwise ROC curve comparison for the logistic regression models trained and tested on 100% of the data sample. (**a**) Means and irregularities of the 6 time intervals condition (MI6_100) compared to mean and irregularity of the whole day condition (MID_100); (**b**) mean and irregularity of the whole day condition (MID_100) compared to repeated measures of consumption in the 6 time intervals condition (RM_100); (**c**) repeated measures of consumption in the 6 time intervals condition (RM_100) compared to means and irregularities of the 6 time intervals condition (MI6_100).

The 0.5 cutoff point worsened sensitivity and NPV, while improving specificity and PPV, meaning that the models became very accurate at identifying the healthy BMI individuals (non-overweight). The sensitivity at the 0.5 cutoff point, however, was low at 51.0% for MID, 51.7% for MI6, and 52.7% for RM, indicating that the models failed to identify nearly half of overweight cases. When it comes to the models at the optimal cutoff point, all of the classification metrics were at a satisfying level, except for PPV, which was 53.4% for MID, 56.3% for MI6, and 57.1% for RM, which means a large proportion of the cases classified as overweight had normal weight in reality. Sensitivity was much higher than for the 0.5 cutoff point, above 70% for all of the three models (Figure 1), while specificity lowered to an acceptable level, reflecting the classic trade-off between specificity and sensitivity. The optimal cutoff point for the MI6 model was much lower than for the other two, which might be why all of its classification metrics scored either the lowest or the highest, always standing out amongst the other two models.

On the pairwise comparisons of the ROC curves for logistic regression models (Figure 1), trained and tested on 100% of the data sample, it is visible that the ROC curves of the models considering 6 time intervals (MI6 and RM) slightly diverge from the ROC curve of the mean and irregularity of the whole day intake (MID). The test of the hypothesis that the area under MI6 ROC (with AUC = 0.7909) and the area under MID ROC (with AUC = 0.7850) are equal, returned a *p*-value of 0.0086 (Table A1), which makes the difference between them highly statistically significant despite the small difference. Similarly, the comparison of the ROC curve for RM (AUC = 0.7923) and for MID returned a *p*-value of 0.0072, which makes the difference between them significant as well (Table A2). Between the ROC curves of MI6 and RM, there was no significant difference (Table A3). All of the *p*-values for pairwise comparisons of the ROC curves are displayed in Appendix A. The AUCs of all of the conditions were above 0.75, which means that the models predicted overweight significantly better than at random, which would result in an AUC of 0.5. They also performed better than models, which did not consider any demographic and behavioural variables beside the energy-related ones, which had AUCs of 0.6146 for RM, 0.6115 for MI6, and 0.5259 for MID.

#### 3.1.2. Logistic Regression Models Trained on 70% and Tested on the Remaining 30% of the Data Sample

After randomly dividing the dataset into a training set and a testing set, the classification metrics computed on the test sample scored visibly lower. The optimal cutoff points in this case were 0.33 for MI6, 0.29 for MID, and 0.32 for RM. The sensitivity at the 0.5 cutoff point (Table 3), which was already low when training and testing at 100% of the data sample, became even lower. It was 45.2% for RM, 44.0% for MI6, and 44.4% for MID, which means more than half of overweight cases were missed. At the 0.5 cutoff point, the RM model performed the best across all of the classification metrics, MID came after it, and MI6 performed the worst; however, the differences were small. The MID model performed best with respect to sensitivity and NPV for the optimal cutoff point (Table 4), but had the highest error rate (31.3% compared to 29.8% for both MI6 and RM).

**Figure 2:**
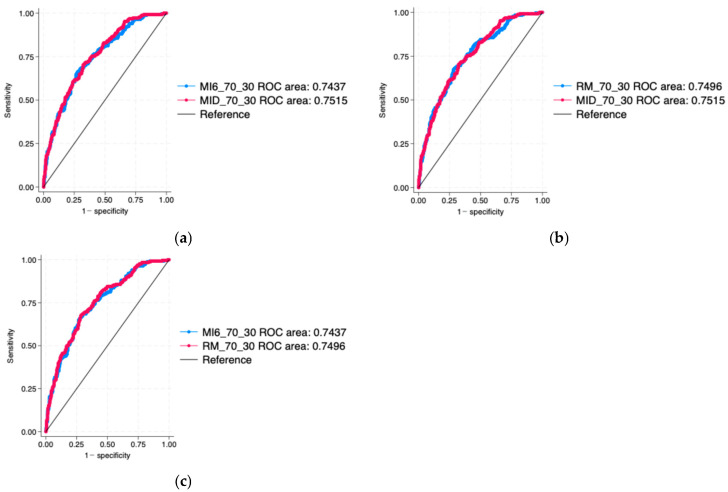
Pairwise ROC curve comparison for the logistic regression models trained on 70% and tested on 30% of the data sample. (**a**) Means and irregularities of the 6 time intervals condition (MI6_70_30) compared to mean and irregularity of the whole day condition (MID_70_30); (**b**) mean and irregularity of the whole day condition (MID_70_30) compared to repeated measures of consumption in the 6 time intervals condition (RM_70_30); (**c**) repeated measures of consumption in the 6 time intervals condition (RM_70_30) compared to means and irregularities of the 6 time intervals condition (MI6_70_30).

The areas and ROC were no longer statistically significantly different. In particular, the condition considering the mean and irregularity of the whole day even had the largest AUC (AUC = 0.7437 for MI6, AUC = 0.7496 for RM, AUC = 0.7515 for MID, Figure 2); however, the differences were small and not significantly different for any of the comparisons (*p*-value = 0.1464 for comparison of MID with MI6, as seen in Table A4, and *p*-value = 0.7489 for comparison of MID with RM, as seen in Table A5, and *p*-value of 0.1989 for comparison between MI6 and RM, as seen in Table A6).

#### 3.1.3. Cross-Validated Logistic Regression Models

**Figure 3:**
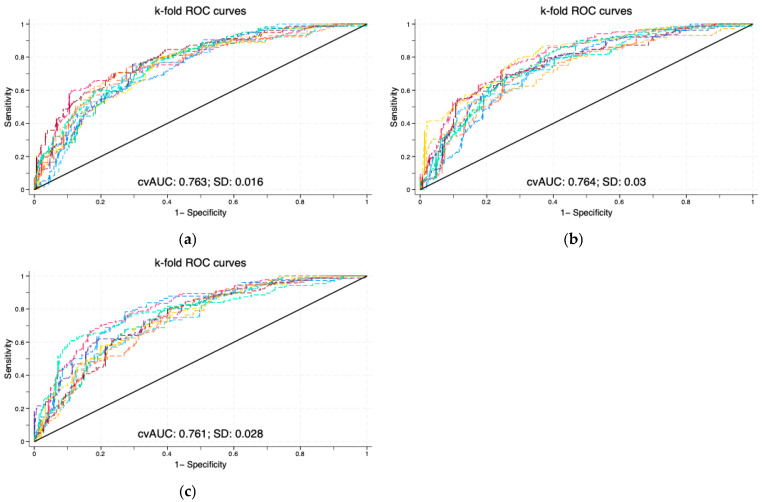
Visualization of the ROC curves for the cross-validated logistic regression models, generated at 10-folds for (**a**) repeated measures of consumption in the 6 time intervals (RM); (**b**) means and irregularities of the 6 time intervals condition (MI6); (**c**) mean and irregularity of the whole day condition (MID).

The 10-fold cross-validated mean AUCs of the MI6 and RM considering chrononutrition information were virtually the same as the mean and irregularity of the whole day MID (mean AUC of 0.763 for RM, 0.764 for MI6 compared to 0.761 for MID). In particular, their 95% confidence intervals were overlapping (Table A10, Table A11 and Table A12). However, the repeated measures model (RM) had a mean standard deviation much lower than the other models (0.016 for RM; 0.030 for MI6; 0.028 for MID) (Figure 3). This suggests that providing the specific amount of energy consumed daily in the 6 time intervals produces a more stable model compared to the models that considered only the average of food intake across 3 days, whether with or without the partition by time of the day.

The logistic regression results based on inclusion of all socio-demographic variables were numerically and substantively very similar to the ones obtained by stepwise forward regression.

### 3.2. Random Forest Models

The classification metrics for the random forest models performed most similarly to the classification metrics of the logistic regression models trained, like RFs, on 70% and tested on 30% of the data sample.

None of the three models had a superior performance across all metrics. At the 0.5 cutoff point, the sensitivity for MID was much higher than for the other two models (46.6% for MID comparing to 38.6% for MI6 and 39% for RM) (Table 5). MID also performed better when it comes to NPV (75.6%); however, in this case, the difference was not as large (MI6 scored 73.2% and RM scored 73.6%).

Sensitivity scores were particularly low at the 0.5 cutoff point; more than half of overweight participants were not identified by the models, as a lot of false negatives were generated.

At the 0.5 cutoff, specificity was higher for the models, which considered 6 time intervals, compared to the one considering the day as a whole; however, it was high for all three models, which means they all correctly identified the vast majority of subjects who were not overweight. PPV was also better for the models using time intervals (Table 5).

The differences in the classification metrics for the two models considering time intervals were generally very small at the 0.5 cutoff; only for PPV was the difference a bit larger (63.0% for RM and 60.7% for MI6).

At the optimal cutoff point, which was *p* = 0.363 for RM, *p* = 0.391 for MI6, and *p* = 0.360, the metrics were similar for MID and RM; MI6 differed more from them and had the best error rate performance (Table 6). The sensitivity generally improved at the optimal cutoff point; the RM model scored the highest in this case (71.2% compared to 69.1% for MID and 64.0% for MI6). The specificity for MI6 was 75.6%, followed by MID (69.2%) and RM (68.6%). The values for PPV and NPV were more similar, with difference lower than 4% between any of the models (Table 6).

**Figure 4:**
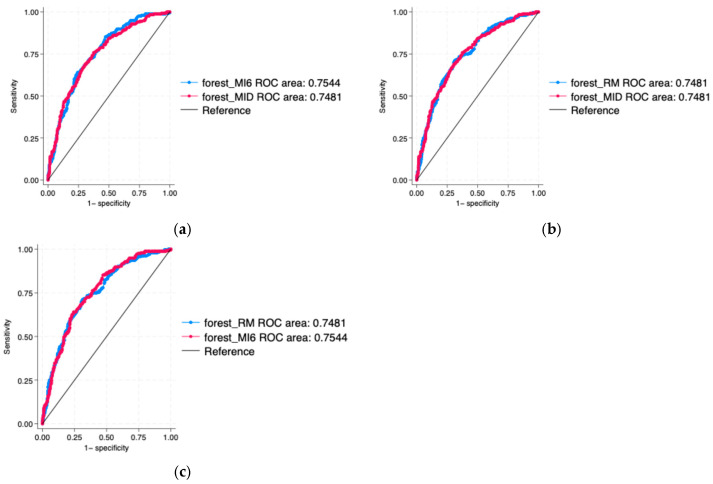
Pairwise ROC curve comparison for the random forest models. (**a**) Means and irregularities of the 6 time intervals (forest_MI6) compared to mean and irregularity of the whole day condition (forest_MID); (**b**) mean and irregularity of the whole day (forest_MID) compared to repeated measures of consumption in the 6 time intervals condition (forest_RM); (**c**) repeated measures of consumption in the 6 time intervals condition (forest_RM) compared to means and irregularities of the 6 time intervals condition (forest_MI6).

When it comes to the ROC curves of random forest models, they were all similar. The MI6 model had the highest AUC = 0.7544, followed by RM and MID models, which both had an AUC of 0.7481. None of the pairwise comparisons (Figure 4) showed a significant difference(*p*-value = 0.511 for MI6 and MID; 0.997 for RM and MID; 0.425 for RM and MI6 comparison), and their 95% confidence intervals were overlapping (Table A7, Table A8 and Table A9). Hence, including the intake data from the 6 time intervals did not change the discriminatory accuracy of the models, which is consistent with the finding for the 30% test set obtained by logistic regression.

The random forest results also come with a ranking of variable importance [23], from which it appeared that some variables consistently featured highly (Table A25, Table A26 and Table A27) across all models. In particular, the energy variables had the highest importance for all three conditions. Except for the energy variables, those which always had relatively high importance were administrative region, the type of area (urban/rural), age, and the geographical area of Italy. For RM and MI6, the energy variables, containing information about energy intake earlier in the day, had the highest importance.

### 3.3. Comparing Models Including Only Energy Variables

To gain more insight in the relevance of chrononutrition information for predictive models, which overall showed a somewhat superior performance for RM, we also evaluated the added value of stratification of energy intake by time of day after removing the influence of the socio-demographic and behavioural predictors, i.e., comparing the three prediction models purely based on energy intake variables. When using the 100% sample for training and testing, AUC = 0.615 for RM, AUC = 0.612 for MI6, and AUC = 0.526 for MID (*p* ≤ 0.001 for comparison test between MID and either RM or MI6, and no significant difference between RM and MI6 model with *p* = 0.657) (Table A13, Table A14 and Table A15). This statistical comparison remained qualitatively unchanged when training on 70% and testing on 30% of the sample, with AUC = 0.618 for RM, AUC = 0.622 for MI6, and AUC = 0.507 for MID (*p* ≤ 0.001 for comparison test between MID and either RM or MI6, and no significant difference between RM and MI6 model with *p* = 0.774) (Table A16, Table A17 and Table A18). Again, when using only energy variables, 10-fold cross-validated logistic regression led to consistent results, in that the average AUC = 0.594 for RM, AUC = 0.597 for MI6, and AUC = 0.523 for MID, with non-overlapping confidence intervals between MID and either RM or MI6 models, which in turn were completely overlapped (Table A22, Table A23 and Table A24).

Finally, the random forest models also showed superior results of the models with chrononutrition information with AUC = 0.5873 for RM, AUC = 0.5880 for MI6, and AUC = 0.5020 for MID with significant differences between RM and MID (*p*-value of 0.0050), as well as MI6 and MID (*p*-value of 0.0049) (Table A19 and Table A20). There was no significant difference between RM and MI6 (*p*-value of 0.9720) (Table A21).

## 4. Discussion

The primary finding of this study was that the inclusion of chrononutrition variables in both logistic regression and random forest models did not result in a statistically or clinically meaningful improvement in the discriminatory accuracy for overweight status beyond that achieved using total daily energy intake. This was observed despite a modestly superior performance of models incorporating repeated dietary measures over three days. Importantly, this conclusion concerned the models that were adjusted for socio-demographic factors, such as age and sex, as well as behavioural predictors, including physical activity and other lifestyle variables.

In contrast, when overweight prediction relied exclusively on dietary information, there was a substantial improvement in discriminatory accuracy across chrononutritional-modelling approaches compared to using just total energy intake over the day. Taken together, these findings suggest that certain socio-demographic and behavioural characteristics may inadvertently capture information related to temporal patterns of eating, thereby diminishing the apparent added value of explicit chrononutrition variables when such covariates are included. However, in the absence of some of these covariates, the additional information provided by timing of energy intake—whether derived from multiple diary days analysed separately or summarised using average intake and irregularity indices—significantly enhanced the discriminatory accuracy for overweight status.

Within the context of observational research, disentangling the physiological effects of circadian regulation from behavioural patterns of eating remains inherently challenging. Indeed, both the timing of eating [24,25,26,27] and markers of circadian disruption [28,29] have been consistently associated with overweight and obesity, yet distinguishing their independent contributions in observational settings is difficult, as eating behaviour is closely intertwined with social, behavioural, and lifestyle factors. This complexity provides an important lens through which to interpret the present findings, in which chrononutrition variables did not meaningfully improve model discrimination once socio-demographic and behavioural covariates were included. Experimental work offers critical insight into these mechanisms. Using a forced desynchrony protocol to separate endogenous circadian regulation from behavioural influences related to time awake and fasting–feeding schedules, Barker and colleagues demonstrated that caloric intake in adolescents is governed by two distinct yet interacting processes: intrinsic circadian control and behavioural regulation linked to the waking cycle [30]. Under tightly controlled conditions, the endogenous circadian system exerted a robust influence on energy intake, favouring greater consumption during the late circadian afternoon and reduced intake during the circadian morning, independent of meal timing, fasting duration, or environmental cues. In parallel, behavioural factors exerted an independent effect, with higher intake earlier after awakening and a progressive decline across the waking episode. Importantly, adolescents with overweight or obesity exhibited both a delayed and attenuated circadian rhythm of caloric intake and a flatter distribution of intake across the waking day, despite preserved central circadian timing as assessed by melatonin. This pattern suggests that excess weight in youth may be associated not with overt disruption of the circadian clock itself, but rather with diminished circadian control over eating behaviour and increased influence of non-circadian drivers, which may weaken temporal constraints on eating and promote later, more evenly distributed caloric intake linked to adverse metabolic outcomes.

Barker and colleagues further provided a quantitative comparison of the relative strength of circadian and behavioural influences on intake by expressing both effects as a proportion of the mesor [30]. They showed that the circadian peak-to-trough amplitude of caloric intake accounted for approximately 34% of the mesor, whereas the difference between the first and last meals of the waking episode accounted for a larger proportion (49%), indicating a stronger apparent behavioural influence when analyses included the longest fasting interval. However, when behavioural effects were examined under conditions of equivalent fasting duration—by restricting comparisons to meals preceded by the same 3 h fasting interval—the magnitude of behavioural variability (31% of the mesor) closely matched the circadian peak-to-trough amplitude. This finding indicates that circadian and behavioural influences on energy intake are of comparable magnitude when confounding by fasting duration is minimized, and that the larger behavioural effect observed across the full waking episode is partly driven by differences in preceding fast length rather than time awake alone. Notably, the balance between circadian and behavioural contributions varied by weight status, with adolescents with obesity showing the smallest divergence between the two, driven largely by a flatter behavioural distribution of caloric intake rather than marked differences in circadian rhythmicity.

Together, this experimental evidence helps contextualise the present modelling results and provides a plausible explanation for why explicit chrononutrition variables offered limited additional discriminatory value when rich socio-demographic and behavioural covariates were included. Measures such as physical activity, dieting behaviour, and other lifestyle factors may implicitly capture information related to both behavioural eating patterns and circadian alignment. Conversely, in the absence of such covariates, timing of energy intake—particularly when represented using repeated daily measures that preserve day-to-day variability—may serve as a proxy for underlying behavioural and circadian regulation, thereby substantially improving discrimination of overweight status. These findings reinforce the importance of distinguishing circadian from behavioural determinants of eating behaviour, and suggest that both predictive models and interventions targeting meal timing may need to consider not only when individuals eat, but also the extent to which circadian regulation of energy intake is preserved. The influence of socio-demographic variables on model performance is particularly relevant when interpreting the limited incremental value of chrononutrition variables observed in this study. Variables such as sex may act as strong predictors in their own right and may also proxy underlying biological and behavioural processes related to timing of eating and obesity risk [31]. Indeed, emerging evidence suggests that the relationship between circadian alignment, meal timing, and adiposity differs between females and males, with sex-specific associations observed for body fat distribution, cardiometabolic risk, and metabolic regulation. In predictive models that include sex alongside behavioural covariates, some of the information captured by chrononutrition variables may therefore be indirectly accounted for, reducing their apparent added value. Importantly, our outcome measure focused on overall overweight status and did not include indices of fat distribution or ectopic adiposity, which are known to differ by sex and to be differentially associated with circadian disruption and eating timing. As a result, potential sex-specific effects of meal timing on regional fat deposition may not have been fully captured by the models. These considerations suggest that the contribution of chrononutrition to overweight prediction may be more pronounced for outcomes that better reflect sex-dependent patterns of adiposity or metabolic risk, and that future prediction models may benefit from incorporating more granular body composition measures alongside sex-stratified analyses to better disentangle behavioural and circadian pathways.

Overall, our findings are consistent with the findings of Di Traglia et al. (2024) [5]. Accordingly, the authors investigated whether using data on timing of eating with deep learning prediction models and with a linear regression model enhances the prediction of BMI, compared to models containing only energy intake from the whole day. They found that data on timing of eating did not enhance the performance of the deep learning models. The authors argued that dividing the energy intake by meal timing introduced unnecessary noise in the learning process. When it came to linear regression, the model with chrononutrition information performed slightly better, albeit not statistically significantly. Our findings align with the latter study, despite using different prediction models and using overweight status as a categorical variable, instead of a continuous BMI variable.

This research has some limitations, in that it was conducted on data collected 20 years ago. During this time, the dietary patterns and day-to-day lifestyle-related activities of the Italian population could have changed, which may also affect the relevance and predictive power of the model. A replication of this study, using more recent data (like INRAN-SCAI 2018–2020 [32]) is warranted to assess the influence of more recent dietary practices related to chrononutrition like intermittent fasting, a type of time-restricted eating that has become more prevalent in the last decade. Further limitations are due to the limited number of days of data collection, which implies that elevated within-subject random error [33] may have affected the chrononutritional variables, resulting in reduced AuROCs. Moreover, the data used were self-reported, which may have introduced problems like incorrect recall of information or errors in measuring the food quantity by the participants, potentially leading to bias. In particular, only if misclassification of intake was non-differential by timing of eating, we might assume a similar impact on prediction for all models we considered. Again, it is arguable whether the data of food intake in specific day times may be more or less affected by misreporting and in comparison to total daily energy intake, though some research suggests that breakfast is better-reported compared to other meals and snacks [34]. The latter, especially late/night ones, may be less accurately reported for practical and conformity reasons. Another element to consider is the tendency of younger adults’ metabolism to be shifted towards later chronotypes [7,35], which may warrant a stratified analysis by age groups.

This study is also limited by the fact that the cross-sectional outcome may not properly capture chrononutrition effects related to circadian alignment and energy regulation over time, as it lacks explicit information on habitual eating window/fasting duration and chronotype. Hence, for future research, a large prospective study could be set up to assess, longitudinally rather than cross-sectionally, the power of chrononutritional habits to predict overweight and obesity, including dietary assessments at distal time points, after an initial health assessment of the participants, and of their chronotype. Such a study would allow one to adopt a causal approach including appropriate control for confounding factors, and so that the potential causal relationship might also be elucidated between timing and regularity of eating and overweight/obesity status.

## 5. Conclusions

To summarize, this study provides a good insight into how the performance of different prediction models, logistic regression and random forest, for predicting overweight status, is influenced by providing meal timing information compared to using the energy intake summary from the whole day only. When including both nutritional and demographic/behavioural predictors, this research only found an improvement in the accuracy of overweight status prediction in the logistic regression models when the models were applied on the same data they were tested on, which made them not suitable for generalization due to likely overfitting. In fact, the models using chrononutrition information had better performance than the models that only used the summary of energy intake from the whole day when only energy variables were included as predictors, whilst when additional socio-demographic variables were included, chrononutrition data did not improve discriminatory accuracy, whether using classic statistical or machine learning methodology. These results indicate that further research is needed to assess the relevance and quality of timing of energy intake, and its relation to other lifestyle variables also in order to optimise the design, data collection, and dietary assessment of future nutrition surveys studies. Additional refinement may come from larger prospective datasets where obesity rather than overweight may be the outcome, as well as from more modern tools to draw objective measures of food intake during 24 h. Longitudinal analysis could be useful to confirm or further disentangle the direction of causality in the relationship between the timing and regularity of eating and increased body weight.

## Figures and Tables

**Table 1:** Logistic regression models trained and tested on 100% of the data sample, at the optimal cutoff point.

Condition	Sensitivity	Specificity	PPV ^1^	NPV ^2^	Error Rate
Mean and irregularity of the whole day (MID)	71.1%	72.0%	57.4%	82.5%	28.3%
Means and irregularities of 6 time intervals (MI6)	80.7%	64.2%	54.4%	86.3%	30.1%
Repeated intake measures for 6 time intervals (RM)	70.6%	73.4%	58.4%	82.5%	27.6%

^1^ PPV = positive predictive value. ^2^ NPV = negative predictive value. The specificity, sensitivity, positive predictive value, negative predictive value, and error rate, obtained from the logistic regression models, trained and tested on 100% of the data sample, at an optimal predicted probability cutoff point, considering the three conditions: mean and irregularity of energy intake during the whole day (MID); means and irregularities of the energy intake from 6 time intervals (MI6); repeated measures of the energy intake, in the time intervals across the 3 days of the survey (RM).

**Table 2:** Logistic regression models trained and tested on 100% of the data sample, at 0.5 cutoff point.

Condition	Sensitivity	Specificity	PPV ^1^	NPV ^2^	Error Rate
Mean and irregularity of the whole day (MID)	51.0%	86.4%	66.6%	76.9%	25.8%
Means and irregularities of 6 time intervals (MI6)	51.7%	86.5%	66.9%	77.2%	25.6%
Repeated intake measures for 6 time intervals (RM)	52.7%	87.0%	68.1%	77.6%	24.9%

^1^ PPV = positive predictive value. ^2^ NPV = negative predictive value. The specificity, sensitivity, positive predictive value, negative predictive value, and error rate, obtained from the logistic regression models, trained and tested on 100% of the data sample, at a *p* = 0.5 cutoff point, considering the three conditions: mean and irregularity of energy intake during the whole day (MID); means and irregularities of energy intake from 6 time intervals (MI6); repeated measures of energy intake, in the time intervals across the 3 days of the survey (RM).

**Table 3:** Logistic regression models trained on 70% and tested on 30% of the data sample, at 0.5 cutoff point.

Condition	Sensitivity	Specificity	PPV ^1^	NPV ^2^	Error Rate
Mean and irregularity of the whole day (MID)	44.4%	86.0%	64.5%	73.0%	29.1%
Means and irregularities of 6 time intervals (MI6)	44.0%	84.4%	61.8%	72.5%	30.3%
Repeated intake measures for 6 time intervals (RM)	45.2%	86.7%	66.1%	73.5%	28.4%

^1^ PPV = positive predictive value. ^2^ NPV = negative predictive value. The specificity, sensitivity, positive predictive value, negative predictive value, and error rate, obtained from the logistic regression models, trained on 70% and tested on 30% of the data sample, at a 0.5 cutoff point, considering the three conditions: mean and irregularity of energy intake during the whole day (MID); means and irregularities of the energy intake from 6 time intervals (MI6); repeated measures of the energy intake, in the time intervals across the 3 days of the survey (RM).

**Table 4:** Logistic regression models trained on 70% and tested on 30% of the data sample, at the optimal cutoff point.

Condition	Sensitivity	Specificity	PPV ^1^	NPV ^2^	Error Rate
Mean and irregularity of the whole day (MID)	70.4%	67.7%	55.5%	80.0%	31.3%
Means and irregularities of 6 time intervals (MI6)	66.0%	72.5%	57.9%	78.9%	29.8%
Repeated intake measures for 6 time intervals (RM)	67.6%	71.6%	57.7%	79.4%	29.8%

^1^ PPV = positive predictive value. ^2^ NPV = negative predictive value. The specificity, sensitivity, positive predictive value, negative predictive value, and error rate, obtained from the logistic regression models, trained on 70% and tested on 30% of the data sample, at an optimal cutoff point, considering the three conditions: mean and irregularity of energy intake during the whole day (MID); means and irregularities of the energy intake from 6 time intervals (MI6); repeated measures of the energy intake, in the time intervals across the 3 days of the survey (RM).

**Table 5:** Random forest models with a 0.5 cutoff point.

Condition	Sensitivity	Specificity	PPV ^1^	NPV ^2^	Error Rate
Mean and irregularity of the whole day (MID)	46.6%	85.7%	62.9%	75.6%	27.6%
Means and irregularities of 6 time intervals (MI6)	38.6%	87.0%	60.7%	73.2%	29.5%
Repeated intake measures for 6 time intervals (RM)	39.0%	88.1%	63.0%	73.6%	28.7%

^1^ PPV = positive predictive value. ^2^ NPV = negative predictive value. The specificity, sensitivity, positive predictive value, negative predictive value, and error rate, obtained from the random forest models, at a 0.5 cutoff point, considering the three models: mean and irregularity of energy intake during the whole day (MID); means and irregularities of the energy intake from 6 time intervals (MI6); repeated measures of the energy intake, in the time intervals across the 3 days of the survey (RM).

**Table 6:** Random forest models with an optimal cutoff point.

Condition	Sensitivity	Specificity	PPV ^1^	NPV ^2^	Error Rate
Mean and irregularity of the whole day (MID)	69.1%	69.2%	53.8%	81.2%	30.8%
Means and irregularities of 6 time intervals (MI6)	64.0%	75.6%	57.6%	80.2%	28.4%
Repeated intake measures for 6 time intervals (RM)	71.2%	68.6%	54.0%	82.1%	30.5%

^1^ PPV = positive predictive value. ^2^ NPV = negative predictive value. The specificity, sensitivity, positive predictive value, negative predictive value, and error rate, obtained from the random forest models, at an optimal cutoff point, considering the three models: mean and irregularity of energy intake during the whole day (MID); means and irregularities of the energy intake from 6 time intervals (MI6); repeated measures of the energy intake in the time intervals across the 3 days of the survey (RM).

## Data Availability

Data can be downloaded from the FAO/WHO GIFT platform: Data FAO/WHO GIFT|Global Individual Food Consumption Data Tool (https://www.fao.org/gift-individual-food-consumption/data/en, accessed on 12 February 2026).

## Abbreviations

The following abbreviations are used in this manuscript:

PPV	Positive Predictive Value
NPV	Negative Predictive Value
ROC	Receiver Operating Characteristic
MI6	Means and Irregularities of 6 time intervals
MID	Means and Irregularity of the whole Day
RM	Repeated Measures of energy intake in 6 time intervals
GLP-1	Glucagon-Like Peptide 1
BMI	Body Mass Index

## Appendix A

**Table A1:** The metrics obtained from comparing the ROC curves of logistic regression models, trained and tested on 100% of the data sample, for the conditions of means and irregularities of 6 time intervals (MI6_100); mean and irregularity of the whole day (MID_100).

Condition	Number of Observations	ROC Area	Standard Error	95% Confidence Interval
MI6_100	2276	0.7909	0.0097	0.77193–0.80979
MID_100	2276	0.7850	0.0097	0.76595–0.80414
H0: area(MI6_100) = area(MID_100)				
chi2(1) = 6.90			Prob > chi2 = 0.0086	

**Table A2:** The metrics obtained from comparing the ROC curves of logistic regression models, trained and tested on 100% of the data sample, for the conditions of repeated measures of 6 time intervals (RM_100); mean and irregularity of the whole day (MID_100).

Condition	Number of Observations	ROC Area	Standard Error	95% Confidence Interval
RM_100	2276	0.7923	0.0096	0.77342–0.81114
MID_100	2276	0.7850	0.0097	0.76595–0.80414
H0: area(RM_100) = area(MID_100)				
chi2(1) = 7.23			Prob > chi2 = 0.0072	

**Table A3:** The metrics obtained from comparing the ROC curves of logistic regression models, trained and tested on 100% of the data sample, for the conditions of repeated measures of 6 time intervals (RM_100); mean and irregularity of 6 time intervals (MI6_100).

Condition	Number of Observations	ROC Area	Standard Error	95% Confidence Interval
RM_100	2276	0.7923	0.0096	0.77342–0.81114
MI6_100	2276	0.7909	0.0097	0.77193–0.80979
H0: area(RM_100) = area(MI6_100)				
chi2(1) = 0.55			Prob > chi2 = 0.4596	

**Table A4:** The metrics obtained from comparing the ROC curves of logistic regression models, trained on 70% and tested on 30% of the data sample, for the conditions of means and irregularities of 6 time intervals (MI6_70_30); mean and irregularity of the whole day (MID_70_30).

Condition	Number of Observations	ROC Area	Standard Error	95% Confidence Interval
MI6_70_30	687	0.7437	0.0192	0.70603–0.78131
MID_70_30	687	0.7515	0.0187	0.71478–0.78820
H0: area(MI6_70_30) = area(MID_70_30)				
chi2(1) = 2.11			Prob > chi2 = 0.1464	

**Table A5:** The metrics obtained from comparing the ROC curves of logistic regression models, trained on 70% and tested on 30% of the data sample, for the conditions of repeated measures of consumption in 6 time intervals (RM_70_30); mean and irregularity of the whole day (MID_70_30).

Condition	Number of Observations	ROC Area	Standard Error	95% Confidence Interval
RM_70_30	687	0.7496	0.019	0.71228–0.78683
MID_70_30	687	0.7515	0.0187	0.71478–0.78820
H0: area(RM_70_30) = area(MID_70_30)				
chi2(1) = 0.10			Prob > chi2 = 0.7489	

**Table A6:** The metrics obtained from comparing the ROC curves of logistic regression models, trained on 70% and tested on 30% of the data sample, for the conditions of repeated measures of consumption in 6 time intervals (RepeatM_70_30); means and irregularities of 6 time intervals (MI6_70_30).

Condition	Number of Observations	ROC Area	Standard Error	95% Confidence Interval
RM_70_30	687	0.7496	0.019	0.71228–0.78683
MI6_70_30	687	0.7437	0.0192	0.70603–0.78131
H0: area(RM_70_30) = area(MI6_70_30)				
chi2(1) = 1.65			Prob > chi2 = 0.1989	

**Table A7:** The metrics obtained from comparing the ROC curves of random forest models, for the conditions of means and irregularities of the whole day (forest_MID); means and irregularities of 6 time intervals (forest_MI6).

Condition	Number of Observations	ROC Area	Standard Error	95% Confidence Interval
forest_MI6	691	0.7544	0.0188	0.71747–0.79126
forest_MID	691	0.7481	0.0194	0.71006–0.78613
H0: area(forest_MI6) = area(forest_MID)				
chi2(1) = 0.43			Prob > chi2 = 0.5108	

**Table A8:** The metrics obtained from comparing the ROC curves of random forest models, for the conditions of repeated measures of consumption in 6 time intervals (forest_RM); means and irregularities of the whole day (forest_MID).

Condition	Number of Observations	ROC Area	Standard Error	95% Confidence Interval
forest_RM	691	0.7481	0.0193	0.71024–0.78602
forest_MID	691	0.7481	0.0194	0.71006–0.78613
H0: area(forest_RM) = area(forest_MID)				
chi2(1) = 0.00			Prob > chi2 = 0.9974	

**Table A9:** The metrics obtained from comparing the ROC curves of random forest models, for the conditions of repeated measures of consumption in 6 time intervals (forest_RM); means and irregularities of 6 time intervals (forest_MI6).

Condition	Number of Observations	ROC Area	Standard Error	95% Confidence Interval
forest_RM	691	0.7481	0.0193	0.71024–0.78602
forest_MI6	691	0.7544	0.0188	0.71747–0.79126
H0: area(forest_RM) = area(forest_MI6)				
chi2(1) = 0.64			Prob > chi2 = 0.4252	

**Table A10:** Summary statistics of the ROC curves for the cross-validated logistic regression model, generated at 10-folds in the condition of repeated measures of consumption in 6 time intervals.

Cross-Validated (10-Fold) Model for RepeatM			
Mean AUC	Mean Standard Deviation	95% Confidence Interval	Prevalence of Overweight
0.7631	0.0162	0.7045–0.7836	34.60%

**Table A11:** Summary statistics of the ROC curves for the cross-validated logistic regression model, generated at 10 folds in the condition of means and irregularities of 6 time intervals for food consumption.

Cross-Validated (10-Fold) Model for MI6			
Mean AUC	Mean Standard Deviation	95% Confidence Interval	Prevalence of Overweight
0.7642	0.0296	0.7458–0.7886	34.60%

**Table A12:** Summary statistics of the ROC curves for the cross-validated logistic regression model, generated at 10-folds in the condition of mean and irregularity of food consumption of the whole day.

Cross-Validated (10-Fold) Model for MID			
Mean AUC	Mean Standard Deviation	95% Confidence Interval	Prevalence of Overweight
0.7613	0.0275	0.7429–0.7848	34.60%

**Table A13:** The metrics obtained from comparing the ROC curves of logistic regression models, trained and tested on 100% of the data sample, with energy variables only, for the conditions of means and irregularities of 6 time intervals (MI6e_100); mean and irregularity of the whole day (MIDe_100).

Condition	Number of Observations	ROC Area	Standard Error	95% Confidence Interval
MI6e_100	2312	0.6115	0.0123	0.58746–0.63557
MIDe_100	2312	0.5259	0.0128	0.50089–0.55096
H0: area(MI6e_100) = area(MIDe_100)				
chi2(1) = 29.41			Prob > chi2 = 0.0000	

**Table A14:** The metrics obtained from comparing the ROC curves of logistic regression models, trained and tested on 100% of the data sample, with energy variables only, for the conditions of repeated measures of 6 time intervals (RMe_100); mean and irregularity of the whole day (MIDe_100).

Condition	Number of Observations	ROC Area	Standard Error	95% Confidence Interval
RMe_100	2312	0.6146	0.0123	0.59060–0.63866
MIDe_100	2312	0.5259	0.0128	0.50089–0.55096
H0: area(RMe_100) = area(MIDe_100)				
chi2(1) = 31.03			Prob > chi2 = 0.0000	

**Table A15:** The metrics obtained from comparing the ROC curves of logistic regression models, trained and tested on 100% of the data sample, with energy variables only, for the conditions of repeated measures of the 6 time intervals (RMe_100); mean and irregularity of 6 time intervals (MI6e_100).

Condition	Number of Observations	ROC Area	Standard Error	95% Confidence Interval
RMe_100	2312	0.6146	0.0123	0.59060–0.63866
MI6e_100	2312	0.6115	0.0123	0.58746–0.63557
H0: area(RMe_100) = area(MI6e_100)				
chi2(1) = 0.20			Prob > chi2 = 0.6568	

**Table A16:** The metrics obtained from comparing the ROC curves of logistic regression models, trained on 70% and tested on 30% of the data sample, with energy variables only, for the conditions of means and irregularities of 6 time intervals (MI6e_70/30); mean and irregularity of the whole day (MIDe_70/30).

Condition	Number of Observations	ROC Area	Standard Error	95% Confidence Interval
MI6e_70/30	690	0.6219	0.0224	0.57805–0.66579
MIDe_70/30	690	0.5074	0.0234	0.46154–0.55330
H0: area(MI6e_70/30) = area(MIDe_70/30)				
chi2(1) = 15.83			Prob > chi2 = 0.0001	

**Table A17:** The metrics obtained from comparing the ROC curves of logistic regression models, trained on 70% and tested on 30% of the data sample, with energy variables only, for the conditions of repeated measures of consumption in 6 time intervals (RMe_70/30); mean and irregularity of the whole day (MIDe_70/30).

Condition	Number of Observations	ROC Area	Standard Error	95% Confidence Interval
RMe_70/30	690	0.6180	0.0224	0.57413–0.66186
MIDe_70/30	690	0.5074	0.0234	0.46154–0.55330
H0: area(RMe_70/30) = area(MIDe_70/30)				
chi2(1) = 14.24			Prob > chi2 = 0.0002	

**Table A18:** The metrics obtained from comparing the ROC curves of logistic regression models, trained on 70% and tested on 30% of the data sample, with energy variables only, for the conditions of repeated measures of consumption in 6 time intervals (RMe_70/30); means and irregularities of 6 time intervals (MI6e_70/30).

Condition	Number of Observations	ROC Area	Standard Error	95% Confidence Interval
RMe_70/30	690	0.6180	0.0224	0.57413–0.66186
MI6e_70/30	690	0.6219	0.0224	0.57805–0.66579
H0: area(RMe_70/30) = area(MI6e_70/30)				
chi2(1) = 0.08			Prob > chi2 = 0.7738	

**Table A19:** The metrics obtained from comparing the ROC curves of random forest models, with energy variables only, for the conditions of means and irregularities of the whole day (forest_MIDe); means and irregularities of 6 time intervals (forest_MI6e).

Condition	Number of Observations	ROC Area	Standard Error	95% Confidence Interval
forest_MI6e	703	0.5880	0.0219	0.54508–0.63091
forest_MIDe	703	0.5020	0.0226	0.45776–0.54628
H0: area(forest_MI6e) = area(forest_MIDe)				
chi2(1) = 7.90			Prob > chi2 = 0.0049	

**Table A20:** The metrics obtained from comparing the ROC curves of random forest models, with energy variables only, for the conditions of repeated measures of consumption in 6 time intervals (forest_RMe); mean and irregularity of the whole day (forest_MIDe).

Condition	Number of Observations	ROC Area	Standard Error	95% Confidence Interval
forest_RMe	703	0.5873	0.0223	0.54362–0.63104
forest_MIDe	703	0.5020	0.0226	0.45776–0.54628
H0: area(forest_RMe) = area(forest_MIDe)				
chi2(1) = 7.88			Prob > chi2 = 0.0050	

**Table A21:** The metrics obtained from comparing the ROC curves of random forest models, with energy variables only, for the conditions of repeated measures of consumption in 6 time intervals (forest_RMe); means and irregularities of 6 time intervals (forest_MI6e).

Condition	Number of Observations	ROC Area	Standard Error	95% Confidence Interval
forest_RMe	703	0.5873	0.0223	0.54362–0.63104
forest_MI6e	703	0.5880	0.0219	0.54508–0.63091
H0: area(forest_RMe) = area(forest_MI6e)				
chi2(1) = 0.00			Prob > chi2 = 0.9720	

**Table A22:** Summary statistics of the ROC curves for the cross-validated logistic regression model, with energy variables only, generated at 10-folds in the condition of means and irregularities of 6 time intervals for food consumption (MI6e).

Cross-Validated (10-Fold) Model for MI6e			
Mean AUC	Mean Standard Deviation	95% Confidence Interval	Prevalence of Overweight
0.5972	0.0367	0.5620–0.6151	34.60%

**Table A23:** Summary statistics of the ROC curves for the cross-validated logistic regression model, with energy variables only, generated at 10-folds in the condition of mean and irregularity of the whole day (MIDe).

Cross-Validated (10-Fold) Model for MIDe			
Mean AUC	Mean Standard Deviation	95% Confidence Interval	Prevalence of Overweight
0.5232	0.0495	0.4899–0.5426	34.60%

**Table A24:** Summary statistics of the ROC curves for the cross-validated logistic regression model, with energy variables only, generated at 10-folds in the condition of repeated measures of consumption in 6 time intervals (RMe).

Cross-Validated (10-Fold) Model for RMe			
Mean AUC	Mean Standard Deviation	95% Confidence Interval	Prevalence of Overweight
0.5939	0.0430	0.5594–0.6122	34.60%

## Appendix B

**Table A25:** Variable importance for the random forests model, using repeated measures of energy intake from 6 time intervals (RM).

Variable Importance for RM	
Energy intake on day 1, hours 6–9	1
Energy intake on day 2, hours 6–9	0.99059478
Energy intake on day 3, hours 6–9	0.95347683
Energy intake on day 1, hours 9–12	0.93302697
Energy intake on day 1, hours 12–15	0.89077049
Energy intake on day 2, hours 9–12	0.88768731
Energy intake on day 3, hours 9–12	0.8776063
Energy intake on day 3, hours 12–15	0.87064467
Area type (rural/urban)	0.83974364
Energy intake on day 1, hours 15–19	0.82229423
Energy intake on day 1, hours 19–22	0.79690258
Energy intake on day 2, hours 19–22	0.77628476
Energy intake on day 2, hours 15–19	0.77352393
Energy intake on day 3, hours 19–22	0.76983469
Energy intake on day 3, hours 15–19	0.7674726
Administrative region	0.74243849
Geographical area of Italy	0.67758349
Profession	0.67134168
Alcohol drinking	0.66116462
Energy intake on day 1, hours 22–6	0.6591067
Age	0.65850636
Consumption of fortified products	0.65611106
Hours of light physical activity, daily	0.63523221
Smoking status	0.62210025
Energy intake on day 2, hours 22–6	0.61845732
Energy intake on day 3, hours 22–6	0.61562901
Employment status	0.61388701
Sedentary hours (in a day)	0.60612416
Level of education	0.60031437
Marital status	0.54547996
Hours of sport per week	0.53510274
Use of diet supplements	0.51733416
Type of diet followed	0.5079527
Presence of a special diet	0.49817096
Sex	0.49396085
Restrictive diet in the past year (yes/no)	0.48962103
Duration of the restrictive diet	0.48776229
Eating breakfast	0.47328409
Hours of sport training (in a week)	0.39468854

**Table A26:** Variable importance for the random forests model, using means and irregularities of energy intake in 6 time intervals (MI6).

Variable Importance for MI6	
Mean energy intake, hours 6–9	1
Irregularity for hours 6–9	0.97770277
Irregularity for hours 9–12	0.94976581
Mean energy intake, hours 12–15	0.940975
Mean energy intake, hours 9–12	0.93667431
Irregularity for hours 12–15	0.92268137
Irregularity for hours 15–19	0.86286761
Mean energy intake, hours 19–22	0.85112137
Mean energy intake, hours 15–19	0.84523486
Irregularity for hours 19–22	0.84314241
Area type (rural/urban)	0.81234257
Administrative region	0.77589663
Irregularity for hours 22–6	0.7650263
Age	0.72659601
Mean energy intake, hours 22–6	0.72634736
Profession	0.7067222
Geographical area of Italy	0.70277085
Hours of light physical activity, daily	0.69892279
Consumption of fortified products	0.67562245
Alcohol drinking	0.66960808
Level of education	0.66041701
Smoking status	0.64236364
Sedentary hours (in a day)	0.63016177
Employment status	0.61929993
Hours of sport per week	0.57539642
Marital status	0.57330578
Presence of a special diet	0.56734783
Sex	0.52978376
Type of diet followed	0.5177077
Hours of sport training (in a week)	0.50768953
Restrictive diet in the past year (yes/no)	0.50743394
Use of diet supplements	0.50464027
Duration of the restrictive diet	0.49337999
Eating breakfast	0.45774674

**Table A27:** Variable importance for the random forests model, using means and irregularities of energy intake during the whole day (MID).

Variable Importance for MID	
Mean energy intake during the whole day	1
Irregularity for the whole day	0.95975921
Age	0.8466771
Administrative region	0.83119202
Area type (rural/urban)	0.82301668
Geographical area of Italy	0.7280391
Profession	0.72440065
Hours of light physical activity, daily	0.70842155
Level of education	0.69820176
Consumption of fortified products	0.68663631
Alcohol drinking	0.6691343
Employment status	0.66310932
Smoking status	0.65105691
Sex	0.64080533
Sedentary hours (in a day)	0.63247123
Hours of sport per week	0.62839109
Marital status	0.61605528
Presence of a special diet	0.55102351
Duration of the restrictive diet	0.5486785
Use of diet supplements	0.51505282
Hours of sport training (in a week)	0.49688438
Restrictive diet in the past Restrictive diet in the past year (yes/no)	0.4892421
Type of diet followed	0.45680849
Eating breakfast	0.40659496
